# Heart transcriptome of the bank vole (*Myodes glareolus*): towards understanding the evolutionary variation in metabolic rate

**DOI:** 10.1186/1471-2164-11-390

**Published:** 2010-06-21

**Authors:** Wiesław Babik, Michał Stuglik, Weihong Qi, Marzanna Kuenzli, Katarzyna Kuduk, Paweł Koteja, Jacek Radwan

**Affiliations:** 1 Institute of Environmental Sciences, Jagiellonian University, ul. Gronostajowa 7, 30-387 Krakow, Poland; 2 Functional Genomics Center Zurich, Winterthurerstr. 190, 8057 Zurich, Switzerland

## Abstract

**Background:**

Understanding the genetic basis of adaptive changes has been a major goal of evolutionary biology. In complex organisms without sequenced genomes, de novo transcriptome assembly using a longer read sequencing technology followed by expression profiling using short reads is likely to provide comprehensive identification of adaptive variation at the expression level and sequence polymorphisms in coding regions. We performed sequencing and de novo assembly of the bank vole heart transcriptome in lines selected for high metabolism and unselected controls.

**Results:**

A single 454 Titanium run produced over million reads, which were assembled into 63,581 contigs. Searches against the SwissProt protein database and the ENSEMBL collection of mouse transcripts detected similarity to 11,181 and 14,051 genes, respectively. As judged by the representation of genes from the heart-related Gene Ontology categories and UniGenes detected in the mouse heart, our detection of the genes expressed in the heart was nearly complete (> 95% and almost 90% respectively). On average, 38.7% of the transcript length was covered by our sequences, with notably higher (45.0%) coverage of coding regions than of untranslated regions (24.5% of 5' and 32.7% of 3'UTRs). Lower sequence conservation between mouse and bank vole in untranslated regions was found to be partially responsible for poorer UTR representation. Our data might suggest a widespread transcription from noncoding genomic regions, a finding not reported in previous studies regarding transcriptomes in non-model organisms. We also identified over 19 thousand putative single nucleotide polymorphisms (SNPs). A much higher fraction of the SNPs than expected by chance exhibited variant frequency differences between selection regimes.

**Conclusion:**

Longer reads and higher sequence yield per run provided by the 454 Titanium technology in comparison to earlier generations of pyrosequencing proved beneficial for the quality of assembly. An almost full representation of genes known to be expressed in the mouse heart was identified. Usage of the extensive genomic resources available for the house mouse, a moderately (20-40 mln years) divergent relative of the voles, enabled a comprehensive assessment of the transcript completeness. Transcript sequences generated in the present study allowed the identification of candidate SNPs associated with divergence of selection lines and constitute a valuable permanent resource forming a foundation for RNAseq experiments aiming at detection of adaptive changes both at the level of gene expression and sequence variants, that would facilitate studies of the genetic basis of evolutionary divergence.

## Background

Understanding the genetic basis of adaptive changes has been a major goal of evolutionary biology. So far, complete, comprehensive analyses have been possible only in microorganisms (e.g., [1,2]). The advent of a new generation of massively parallel DNA sequencing technologies (reviewed in [3,4]) brings the promise of rapid progress in understanding the genetic basis of adaptation also in more complex organisms, including mammals [5,6]. The marriage of large-scale selection experiments (reviewed in [7]) with new sequencing technologies appears to be a prospective research strategy to this end.

Even now, whole genome resequencing in most non-model eukaryotes, possessing complex genomes, is not a viable option, due to challenges with assembly in the presence of large amounts of repetitive sequences, and it is unclear whether the situation will improve in the near future [8]. Therefore, researchers have turned to transcriptome analysis as a powerful and universal tool for identification of both variation at the gene expression level and sequence polymorphisms in coding regions. Deep-coverage transcriptome sequencing (RNAseq) enables the developmental stage and/or tissue-specific analysis of the abundance of transcripts as well as detection of sequence variants [9-12]. Thus, a comprehensive characterization of the transcriptional differences between selection regimes in terms of single nucleotide polymorphisms (SNP), splicing variants, transcription start sites and at the level of transcription of individual genes is possible. The design and feasibility of RNAseq experiments, however, depend on the availability of the reference genome to which the short reads from RNAseq experiments are aligned. If the reference genome is not available, which is the case for the majority of non-model eukaryotes, the lack of genomic resources may be circumvented by employing a two-step strategy: i) assemble the transcriptome de novo [13-15], and then ii) use the assembly as a reference to align the short reads from RNAseq experiments. If the initial assembly is performed on sequences derived from multiple individuals, the detection of sequence differences between individuals or populations (e.g., between selection regimes) can be also performed at this stage.

Theoretically, producing both de novo assembly and obtaining information about the levels of transcription would be possible in a single step, although currently available technologies impose serious constrains on such experiments. Technology offering long reads (454/Roche) does not provide enough coverage (ca. 0.5 Gb of sequence data) for detailed expression profiling, while assembling short reads provided in large amounts (> 50 Gb) by Illumina and ABI SOLiD has been notoriously difficult. Thus, de novo assembly using 454 technology or a combination of 454 and shorter read technologies [16], followed by expression profiling using short reads seems a reasonable approach.

When selecting the organ(s) and/or developmental stage(s) for transcriptome characterization one encounters a tradeoff between maximizing the number of distinct transcripts and maximizing coverage of individual transcripts, the two determinants of transcriptome completeness. This tradeoff is likely to remain even when cDNA normalization is used to limit the variation in abundance of transcripts from various genes, simply because the expression of many genes is spatially or temporally restricted. A common practice that maximizes transcript discovery is pooling RNA extracted from multiple tissues and/or developmental stages [13,14]. However, this approach usually comes at the expense of the completeness of individual transcript sequences. While sequences of housekeeping genes, which are highly expressed in most tissues and developmental stages, will be fully reconstructed, genes with low or limited expression might be entirely missed or only patchily covered. In higher eukaryotes, an additional problem is widespread alternative splicing, often tissue-specific [17-19], which may compromise transcriptome assembly. Therefore, the other popular approach is to characterize the transcriptome of a single tissue [20,21].

In the present study, we performed analysis and de novo assembly of the bank vole heart transcriptome using 454/Roche Titanium technology. The bank vole is an important organism in evolutionary, ecological and behavioral studies [22-25]. However, its genome is not available, and, to our knowledge, no genome project for this species is under way. The direct impetus for this study has been a large experimental evolution study using bank voles selected for high aerobic metabolism during locomotor activity [26]. The experiment addresses important questions about the evolution of endothermy and the genetic architecture of intra- and interspecific variation in metabolic rates. Genomic and transcriptomic resources are essential for extracting the maximum amount of information from this large-scale experiment [26]. We decided to concentrate on the transcriptome of a single tissue and selected the heart because its role in aerobic exercise performance is obvious, and differential gene expression in hearts of rats from lines characterized by low and high aerobic capacity has been already reported [27,28]. Association of differential gene expression and aerobic metabolism of the heart has been also shown at the level of individual variation in fish [29].

During the analysis of the bank vole heart transcriptome, we used the genomic resources available for the best characterized model mammal - the laboratory mouse. The bank vole and mouse diverged ca. 20-40 million years ago [30,31]. This moderate level of divergence, although too high for direct mapping of the bank vole short sequencing reads to the mouse genome, makes possible similarity searches of both protein sequences and nucleotide sequences available for mouse. Thus, a more comprehensive evaluation of the completeness of the transcriptome than mere cataloging genes based on similarity to protein sequences is possible. Consequently, in the present paper we not only provide information on the number of protein coding genes but also estimate the completeness of transcripts, including untranslated regions, and evaluate biases with regard to the coverage of various transcript regions. Furthermore, for the first time, we present evidence that a significant part of sequences derived from a "typical" 454 transcriptome study in a nonmodel organism may represent the transcribed non protein-coding parts of genome, for example long noncoding RNAs. Finally, we compare the sequences of transcribed genes between lines selected for high metabolic rate and controls and identify candidate SNPs that underlie the response to selection.

## Results

### Sequencing and assembly

**Figure 1:**
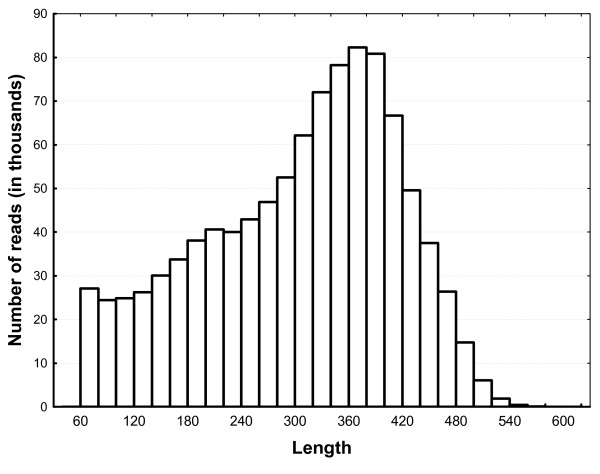
**Length distribution of "cleaned" sequencing reads**. "Cleaning" involved adapter trimming and removal of reads with high similarity to repetitive sequences.

The bank vole cDNA was sequenced in a single 454 Titanium run, which produced a total of 1.109 million reads (351.6 Mb) of an average length of 317 (SD = 127) bp and a median length of 348 bp. Pooled cDNA from four selected lines was sequenced in one half of the picotiter plate and pooled cDNA from four unselected control lines was sequenced in the other half of the plate. All analyses except assessing SNP differences between selected and control lines were performed on the full dataset. After adapter trimming and removal of reads with high similarity to repetitive sequences in RepBase, 1.006 million reads (306.5 Mb) of minimum length 60 bp were used for CAP3 assembly. The detailed statistics of these "cleaned" reads are presented in Table 1 and Fig. 1. It is notable that a substantial number of "cleaned" reads were longer than 400 bp.

**Table 1:** Characteristics of "cleaned" sequencing reads

N reads	1,006,419
N bases	306.5 Mb
Min length (bp)	60
Max length (bp)	593
Mean length (bp)	304.6
SD	109.4
Median length (bp)	325

"Cleaning" involved adapter trimming and removal of reads with high similarity to repetitive sequences.

**Figure 2:**
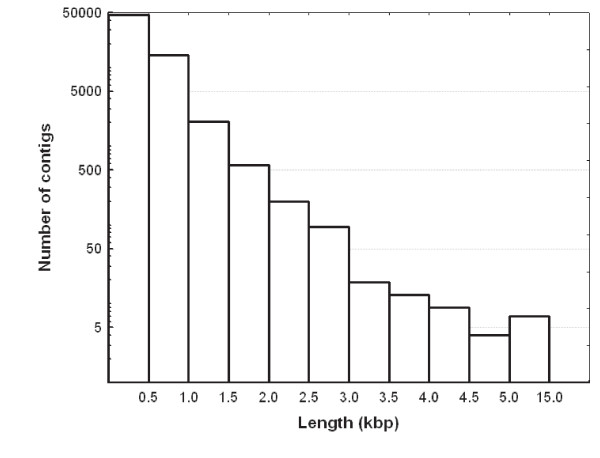
**Length distribution of contigs (note Y axis logarithmic scale)**.

**Figure 3:**
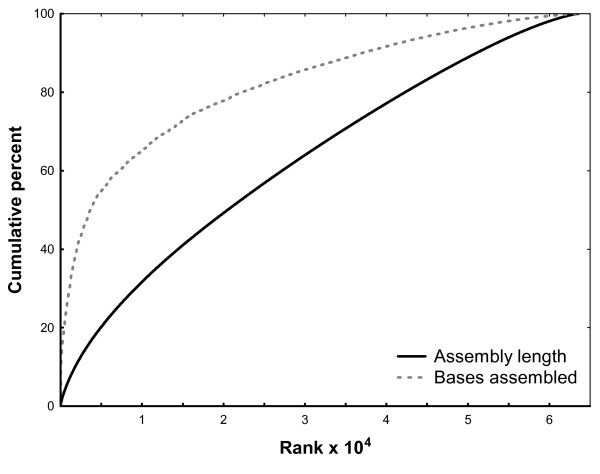
**Cumulative fraction of bases assembled into contigs and the cumulative assembly length**. Contigs are ranked from the longest to the shortest

CAP3 assembling resulted in 63,581 contigs (66.1% of all reads were assembled into contigs) of an average length of 480.6 and a median length of 417 bp; N50 was 477 bp (Table 2). The maximum length of a contig was 13,292 bp, and the length of a substantial number of contigs (349) exceeded 2 kb (Table 3, Fig. 2). The 10% of longest contigs accommodated almost 60% of all assembled bases (Fig. 3). Contigs were composed on average of 10.5 reads (SD = 113.6), however the median number of reads per contig was three (Table 2). Very high coverage of certain contigs should be noted, with the maximum reaching 23,367 reads per contig and the maximum average per base coverage of 2,770. We detected in our trimmed reads 5,763 microsatellite repeats, the majority of them containing dinucleotide motifs (4,772; min. 10 repeat units), followed by tetra- (857; min. 8 units) and trinucleotide repeats (434; min. 8 units).

**Table 2:** Contig statistics

N contigs	63,581
Length (bp)	
Mean	481
SD	300
Median	417
Min	37
Max	13,292
N50	477
Coverage (reads/contig)	
Mean	10.5
SD	113.6
Median	3
Min	2
Max	23,367

**Table 3:** Contig length classes

Length	N contigs
< 500	46,021
500-999	14,565
1,000-1,999	2,646
2,000-2,999	297
3,000-4,999	45
> 5,000	7

### Functional annotation of the transcriptome

Searching the SwissProt database revealed that 18,470 (29.0%) contigs and 44,823 (13.2 %) singletons showed similarity to proteins in the database at an E-value threshold 10^-5 ^(increasing the E-value did not result in a substantial increase in the number of hits). In total, we identified significant similarity to 11,181 genes. Many more sequences exhibited similarity to sequences from the ENSEMBL collection of mouse transcripts (ECMT): 27,283 contigs (42.9%) and 92,957 (27.3%) singletons representing 14,051 ENSEMBL genes (Table 4). Interestingly, a number of contigs (667) and singletons (3,340), that did not have hits in ECMT did have hits in SwissProt. Over 60% of such sequences showed homology to viral or transposon proteins (Table 5). Contigs and singletons without hits in ECMT were blasted against the mouse and rat genomes as well as the AceView [32] nonredundant database of mouse transcripts. A substantial proportion of contigs (58.2%) showed similarity to the mouse or rat genome, and two-thirds of them had hits in both genomes (Table 6). A qualitatively similar picture was obtained for singletons, although the proportion of sequences with hits (33.6%) was lower than for contigs (Table 6). The absolute number of singletons with hits to genomes was higher than the number of singletons with hits to ECMT. A remarkable result is that a large number of sequences (more than one third of the contigs and almost one third of the singletons with similarity to the mouse genome) had hits in the AceView although this database covers only less than 10% of the mouse genome. Thus, sequences that did not match ECMT but matched genomes were highly enriched in sequences known to be transcribed.

**Table 4:** Results of protein and nucleotide database searches

	Swissprot	ECMT
N contigs with hits	18,470	27,283 27.3
% contigs with hits	29.0	42.9
Singletons with hits	44,823	92,957
% singletons with hits	13.2	27.3
N genes identified	11,181	14,051
only in contigs	1,619	1,096
only in singletons	3,326	4,270

ECMT - ENSEMBL Collection of Mouse Transcripts.

**Table 5:** Sequences with hits in Swissprot but not in ECMT

	Contigs	Singletons
N total	667	3340
N with hits to viruses or transposons	416	2152
% with hits to viruses or transposons	62.4	64.4
N unique proteins excluding virus and transposon proteins	157	430

**Table 6:** Contigs and singletons which did not have hit in the ENSEMBL collection of mouse transcripts (ECMT)

		Without hits in ECMT	Hits in genome			Hits in AceView
		
			only mouse	only rat	both	mouse
Contigs	N	36,298	4,247	2,810	14,065	5,948
	%	100	11.7	7.7	38.7	16.4
Singletons	N	340,806	25,423	17,687	71,565	29,305
	%	100	7.5	5.2	21.0	8.6

Results of blast searches against mouse and rat genomes and the AceView collection of mouse transcribed sequences.

We compiled the list of the one hundred most abundant genes, as measured by contigs with the highest per-base coverage (Additional file 1 Table S1). Several conclusions may be drawn from the inspection of this table. Genes for all proteins encoded in mitochondrial and for both mitochondrial ribosomal RNAs were among the high-coverage contigs. A number of nuclear genes encoding mitochondrial proteins were present as well. In contrast, only five genes encoding structural cardiac muscle proteins or proteins involved in the cardiac muscle contraction were detected among the most abundant genes. Overall, although the normalization procedure was successful, as judged from the gel images before and after normalization, the dynamic range of library, expressed as the total number of bases matching a transcript divided by the transcript length, still spanned six orders of magnitude (or five orders of magnitude when the five most highly covered transcripts were excluded).

### Completeness of the transcriptome

To evaluate the completeness of the transcriptome, we checked whether transcripts of all genes normally present in most mammalian cells could be detected. We tested for the presence of genes encoding proteins forming selected macromolecular complexes and genes encoding proteins involved in basic metabolic pathways. In five of six macromolecular complexes and all four evaluated metabolic pathways, all of the involved genes were identified in the bank vole heart transcriptome (Table 7).

**Table 7:** Completeness of selected macromolecular complexes and metabolic pathways

Complex/pathway	Known genes	Detected genes
Macromolecular complexes		
26 S Proteasome	22	22
Chaperonin	8	8
Spliceosome	143	132
Ribosome	79	79
Nuclear Pore Complex	28	28
Respiratory chain complex I	38	38
Metabolic pathways		
Glycolysis	10	10
Gluconeogenesis	10	10
Pentosephospate cycle	7	7
TCA cycle	14	14

We also evaluated the presence of genes that should be expressed in the heart because their products are structural and functional components of the cardiac muscle or are involved in regulation of heart function. We selected five GeneOntology categories related to cardiac muscle organization and contraction: 1) contractile fiber part (GO00044449), 2) myofibril (GO0030016), 3) cardiac myofibril assembly (GO0055003), 4) sacrcomere organization (GO0045214), 5) cardiac muscle contraction (GO0060048), and then compiled a nonredundant list of mammalian genes in these and all children categories and checked whether these genes were detected in our dataset. We excluded from the analysis eleven genes not present in the mouse heart EST library (mainly genes expressed only in skeletal muscles) and detected 129 of the 135 (95.6%) remaining cardiac muscle-related genes in our dataset (Additional file 1 Table S2).

Of the 8,533 UniGenes with assigned gene symbols known to be expressed in the mouse heart (at least one EST), 7,970 of these symbols are present in the ENSEMBL collection of mouse genes. We detected 7,129 (89.4%) of them in our sequences, which indicated that representation of genes expressed in the heart, regardless of their expression levels, was almost complete in our study. This conclusion holds even if we consider all mouse UniGenes, including those with no gene symbol assigned; such UniGenes represent poorly characterized, often weakly expressed transcripts. Blast searches of the bank vole sequences against the entire mouse UniGene database detected 79.9% of the 10,963 UniGenes with expression reported in the heart. On the other hand, sequences similar to 15,630 mouse UniGenes not known to be expressed in the mouse heart were detected, indicating that the expression information in public databases may be very incomplete.

**Figure 4:**
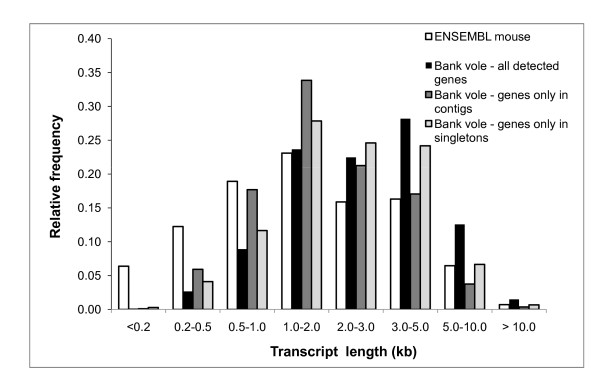
**Comparison of the length distribution of transcripts detected in the bank vole with all mouse transcripts**. The distribution of maximum transcript length (based on the ENSEMBL mouse database) for genes detected in the bank vole compared to the distribution for all mouse genes.

Because two steps of our cDNA preparation procedure involved PCR amplification, a possible bias against detection of long transcripts might have occurred. To evaluate this possibility, we compared the length distribution of transcripts in all mouse ENSEMBL genes (the longest transcript per gene was selected if more than one was available) with the length distribution of transcripts of genes detected in the bank vole. Contrary to the expectation, we found that genes with short transcripts were underrepresented in our experiments, the relative frequencies of genes with transcripts 1-2 kb long were almost identical in ENSEMBL mouse gene collection, and genes with longer transcripts were actually overrepresented in our dataset (Fig. 4). Thus, no bias against the detection of longer transcripts was introduced by our amplification procedures.

**Figure 5:**
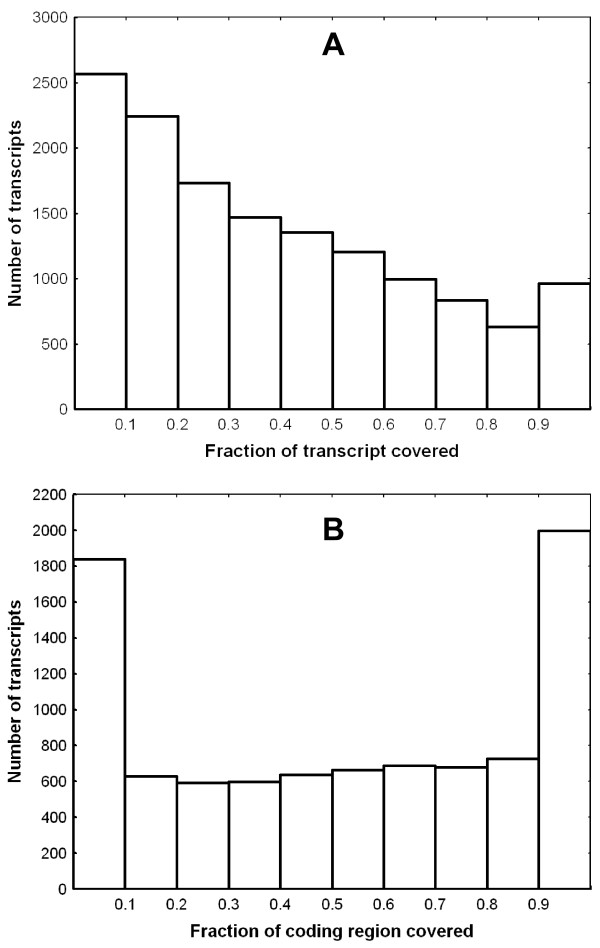
**The completeness of the bank vole transcripts**. Based on the fraction of the mouse ENSEML transcript length covered by aligned bank vole sequences. A) completeness of the total transcript length, B) completeness of the coding regions.

**Figure 6:**
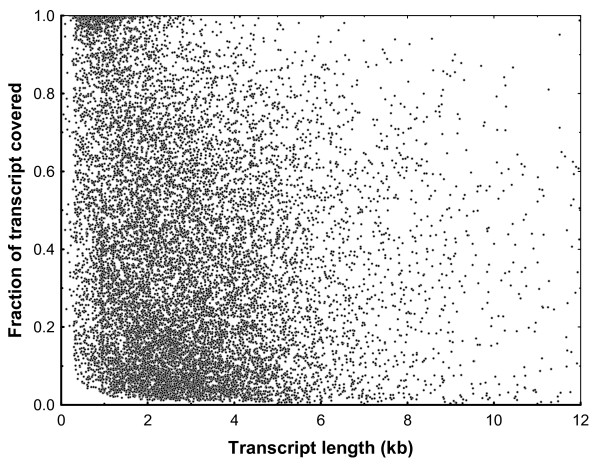
**The relationship between the transcript length and transcript completeness**. The relationship between the mouse transcript length and the fraction of transcript covered by the bank vole sequences. For computation of *R*^2 ^(0.077, *P *< 10^-4^) transcript length was log-transformed and fraction covered arcsin square root transformed, but the plot shows, for clarity non-transformed data and only transcripts up to 12 kb.

**Figure 7:**
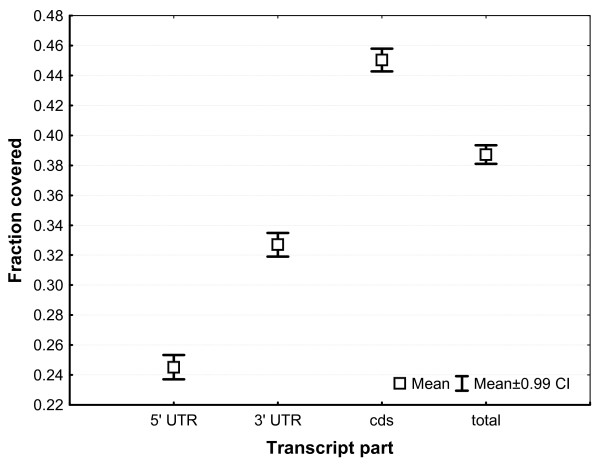
**Completeness of transcripts and transcript regions**. Fractions of the 5' untranslated regions (5' UTR), 3' untranslated regions (3' UTR), coding sequence (cds) and of the total transcript covered by the bank vole sequences.

Another, perhaps more informative, measure of transcriptome completeness is the fraction of the transcript length covered by the bank vole sequences. As the reference we used the data on the transcript length and location of coding sequences from the ECMT (conservatively, the longest transcript per gene was selected if more than one was available). Nearly full (≥90%) transcript length was obtained for 960 (6.8%) transcripts, and for many more (2,148, 15.8% percent of all transcripts with assigned coding sequence (CDS)), an almost complete CDS was identified (Fig. 5). As could have been expected there was a negative correlation between the mouse transcript length and the fraction of transcript covered by the bank vole sequences, although this effect was rather weak (*R*^2 ^= 0.077, *P *< 10^-4^; transcript length log-transformed, fraction covered arcsin-sqrt transformed) (Fig. 6). The mean fraction covered was 0.387 (SD = 0.284). Notably, the coding regions of transcripts had a much higher fraction of their length covered (0.450 ± 0.344) than 3' (0.327 ± 0.350) and 5' (0.245 ± 0.361) UTRs (Fig. 7).

There are at least two alternative explanations for the lower 3' and 5' UTR coverage. It is possible that a bias was introduced during laboratory/sequencing procedures, causing under-representation of cDNA ends both in the primary 454 library and, consequently, in the obtained sequences. On the other hand, under-representation of UTRs may reflect weaker evolutionary conservation of these regions, resulting in a lack of sequence similarity (including possible UTR length differences between mouse and bank vole) to mouse transcripts over a substantial portion of contig/singleton (CS) length. Thus, artifactual "under-representation" of these regions would be caused by sequence divergence in the UTRs beyond the point of blast-detectable similarity and not by the actual bias against UTRs in our sequences. We evaluated these two explanations by analyzing CS mapping to those mouse transcripts that contained the protein coding regions. Assuming that each CS indeed represented a continuous cDNA stretch, for each CS we computed the proportion of its length that did not have significant similarity to the mouse transcript, separately for the parts falling into 5'UTR, CDS and 3'UTR. The proportion was much higher in 5' UTRs (0.311 ± [SE] 0.003) and 3'UTRs (0.360 ± 0.001) than in CDS (0.202 ± 0.001). Thus, weaker evolutionary conservation of untranslated transcript regions substantially contributes to the less complete UTR representation in our study.

**Figure 8:**
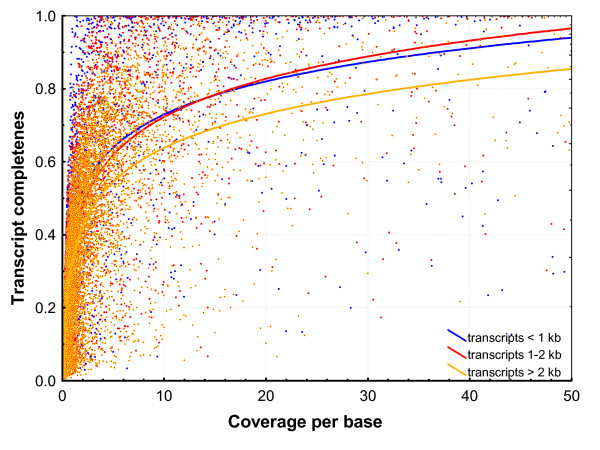
**The relationship between transcript completeness and coverage**. The per base coverage is averaged over the total transcript length. Only coverages up to 50 × are shown. Logarithmic curves were fitted to three subsets of the data: transcripts < 1 kb, 1-2 kb and > 2 kb.

Based on the information about the completeness of the identified transcripts, one may ask how much more sequencing effort would be needed to obtain nearly complete transcript lengths of the majority of genes expressed in the bank vole heart. The relationship between transcript completeness and the per base coverage averaged over the total transcript length (Fig. 8) indicates that, to achieve 75% transcript completeness for transcripts < 2 kb, 12 × coverage is needed, and, for longer transcripts even > 20 × may be required. The coverage obtained in the present study varies widely, but for 75% of transcripts < 2 kb, it was > 0.52 ×. Thus, to achieve the 75% transcript completeness for 75% transcripts < 2 kb with the highest coverage, an additional 22 454 Titanium runs would be theoretically needed, and even more sequencing would be necessary to achieve completeness of longer transcripts. However, 50% completeness of 75% transcripts < 2 kb with the highest coverage would require only three additional runs. The median coverage for transcripts < 2 kb obtained in our study was 1.56×, sufficient to achieve ca. 50% completeness of the half of transcripts (Fig. 8)

### SNP differences between selection regimes

Because most 454 sequencing errors are indels, we analyzed only substitution-type single nucleotide polymorphisms (SNP) in our data. In 19,114 of the SNPs detected by GigaBayes, each variant was present in at least 2 sequencing reads, minimising the impact of sequencing errors [33]. We then compared frequencies of these SNPs between selection regimes. Frequencies of 114 SNPs (in 77 contigs) differed between the selection lines and unselected control at the 10^-4 ^significance level (chi2 test with Yates correction; 2 expected by chance) and 1301 (in 699 contigs representing 560 genes with assigned gene symbol) at the 10^-2 ^level (191 expected by chance). Searches of the second-highest level Gene Ontology categories revealed that genes harboring SNPs that were differentiated between the selection lines at 10^-2 ^level were significantly enriched only for "organelle part" (GO:0044422, P < 10^-4^), and the representation of SNP enriched genes was nonrandom. Further inspection of GO revealed that this was due to the highly significant overrepresentation of genes for mitochondrial proteins (GO:0005739, *P *= 5 × 10^-15^). It should be noted, however, that these genes were overrepresented among the contigs with highest per-base coverage, constituting about half of these genes (Additional file 1 Table S1), which might have made detection of SNPs with significant differences among lineages easier due to the higher coverage.

## Discussion

### Assembly quality

The present study used a third generation of 454 technology (Titanium), which yielded a usable median read length of almost 350 bp. As expected, longer reads produced better assembly, in terms of the average and maximum contig length, than reported in most studies employing the first generation, GS20 [13,34] and second generation, FLX [20,35,36], 454 technologies. Almost three thousand contigs in our dataset exceeded 1,000 bp in length, with the longest contig reaching nearly 14,000 bp, again a substantial improvement in comparison to most studies performed with GS20 and FLX technology. On the other hand, only 66% of all high-quality bases sequenced in the present experiment were assembled into contigs, with 34% remaining as singletons. Similar values were obtained with earlier generations of 454 pyrosequencing. The singleton sequences may either be derived from transcripts of low abundance or represent artifacts from laboratory procedures and/or sequencing. Earlier work showed that most singletons are genuine transcript sequences and therefore it is justified to retain and analyze them further [14]. The results discussed in the following sections provide some explanation of the relatively high proportion of singletons.

### Transcript discovery and functional annotation of the transcriptome

Mining the SwissProt protein database and the ENSEMBL collection of mouse transcripts (ECMT) for sequences similar to those present in our dataset allowed detection of transcripts from a large number of putative genes. More than 11,000 Swiss-Prot proteins and transcripts of over 14,000 ENSEMBL mouse genes produced significant hits. As evidenced by the searches for macromolecular complexes and essential metabolic pathways, our gene detection was practically complete for genes expressed in all tissues. With respect to heart muscle-related gene discovery we found over 95% of 135 mammalian genes assigned GeneOntology categories related to cardiac muscle organization and contraction. Among the most abundant transcripts in our study were those encoded in mitochondrial DNA. This is in accordance with results from SAGE analysis of the adult mouse heart transcriptome, which indicate that the cardiac tissue contains the highest percentage of mitochondrial-genome derived transcripts [37,38]. The estimate of the number of unique transcripts in the adult mouse heart derived from the extrapolation of the results of SAGE experiments exceeds 23,000 [37], and a similar order of magnitude has been suggested for human heart [39]. According to the UniGene, a gene-oriented database of transcribed sequences, the number of transcribed genes in the mouse heart reaches 11,000 (including over 8,500 UniGenes with assigned gene symbols). We detected expression of 80% these genes (89% genes with assigned gene symbols) in the bank vole heart. Moreover, we found in our data sequences similar to over 15,000 other mouse UniGenes, with no heart-confirmed expression. Thus, one the one hand, our gene discovery in the heart appears to be close to complete, but, on the other hand, public resources based mainly on Sanger EST sequencing may be very incomplete with respect to tissue-specific expression.

### Transcript completeness and evaluation of biases

The length of nearly 1,000 transcripts was almost completely (≥90%) covered by our sequences, and when considering only coding regions, this number increased to over 2,000. However, genes with less than 20% of their transcribed length covered constituted almost 34% of all transcripts detected in EMTC, indicating that many transcripts were only patchily reconstructed, a finding further confirmed by the fact that matches to more than 3,000 SwissProt proteins and over 4,000 ECMT were detected only as singletons.

Three factors apparently contributed to the variation in transcript completeness of various genes. First, transcript length was negatively correlated with completeness (Fig. 6), although this factor explained only a minor fraction of variation and many short transcripts were also very incomplete (Fig. 6). Second, normalization was certainly not perfect, with variation still spanning orders of magnitude (see also [40]). Originally rare transcripts would probably also remain rare after normalization, thus producing a low number of reads and resulting in patchy coverage. Third, the sequence divergence from the mouse could have contributed to the less than complete reconstruction of transcripts. We demonstrated this effect by comparing similarity of portions of contigs/singletons matching UTRs and coding regions to mouse sequences. Higher sequence divergence in untranslated regions contributed to the generally lower coverage of UTRs in comparison to the coding parts of transcripts. The lowest coverage of 5'UTRs may also reflect the bias against the 5' end of transcripts expected if polyT primers are used for reverse transcription, although studies differ with regard to the extent of this bias [41,42].

We expected a lower discovery rate for genes with long transcripts because our cDNA preparation method involved PCR with one primer anchored at the 3' end of transcripts. The reverse was true, with a higher proportion of long transcripts detected than observed in the ECMT. However, many long transcripts were detected only as singletons, indicating that average coverage of long transcripts was poor. In a 454 study of the *Arabidopsis *transcriptome, Weber et al. [42] obtained unbiased representations of short (< 1 kb), medium (1-2 kb) and long (> 2 kb) transcripts. In our data virtually no bias was observed for transcripts 1-2 kb long (Fig. 4). The particularly strong underrepresentation of transcripts < 200 bp was probably caused by the RNA extraction method, in which mainly fragments longer than 200 bp bind to a silica membrane.

The two-step approach for transcriptome characterization requires that the expressed sequences be first characterized using long read assembly. In our single Titanium run, we only achieved 45% average completeness for CDS, which may not be enough for effective mapping of short reads to obtain information on expression level. The coverage was lower than expected for two reasons. First, the number of genes we targeted was larger than could have been expected on published information about the number of genes expressed in mouse heart. Second, despite normalization, there was still high variation in coverage among genes, resulting in complete coverage on only 1,000 (or 2,000 if we take only CDS into account) apparently highly expressed genes, and low coverage of most other genes (Fig. 5). Based on our data, we estimated that to achieve a reasonable completeness (≥ 75%) of the three quarters of most abundant (after normalization) transcripts, substantial additional sequencing effort may be needed, rendering the two-step strategy problematic. Our results also suggest that the sequencing effort needed to obtain a reasonable de novo mammalian transcriptome assembly may be higher than suggested by simulations based on sequencing transcriptome of several plants, particularly *Arabidopsis *[16]. Therefore, in organisms with no genomic resources, but that possess close relatives with sequenced-genomes, using these genomes as a reference for mapping short reads (which are becoming longer as technologies mature) might be a more useful strategy. On the other hand, in the absence of related reference genomes, 454 sequencing can still be very useful, taking into account the following considerations. First the coverage per transcript may be improved by increasing normalization efficiency, but differences of an order of magnitude or more would still be expected. Second, the data gathered and assembled in the long read 454 experiments may serve as a useful reference to be filled in with the shorter reads provided abundantly by other sequencing technologies, as suggested previously [16]. The 454 assemblies are particularly likely to be useful in anchoring short contigs derived from short reads. Third, the full lengths of transcripts do not need to be known to perform RNAseq experiments, because one would be able to estimate the expression level of the gene from reads mapping to a known fragment. Fourth, our data on coverage appear to be underestimated due to the divergence from the mouse, and may be missing some UTRs. An only modest increase in the coverage might join these contigs with those representing coding sequences, thus improving both the completeness of transcripts and per-base coverage considerably. Fifth, because we selected the longest transcript per gene, the completeness and per-base coverage values are necessarily conservative. Therefore, we conclude that the approach we present constitutes a reasonable first step towards RNAseq experiments on non-model organisms. In the future, the wide adoption of the pair-end sequencing approach to transcriptome studies with short read technologies may bring rapid progress and become the method of choice for such experiments [43].

### Widespread transcription in noncoding regions?

A notable result emerging from our study is that only a minority of contigs and singletons exhibited sequence similarity to the SwissProt proteins and ENSEMBL mouse transcripts. Therefore, to gain insight into the identity of other sequences we blasted them against the genomes of the mouse and rat. A very large fraction of the bank vole sequences which did not map to ECMT (58.2% of contigs and 33.6% of singletons) had hits in the mouse or rat genome. In a study of another arvicoline rodent, the prairie vole, about one third of random genomic fragments sequenced from the BAC library could have been mapped to the mouse genome [44], a value similar to that obtained for singletons in our study. This could, in principle, indicate a substantial contamination with genomic DNA. However, this possibility seems unlikely given our laboratory procedures, which involved poly-T priming of first strand cDNA synthesis. Instead, we hypothesize that the large number of matches to genomic sequences may be the result of a widespread transcription, known to occur in most eukaryotic genomes, including mouse [45-49]. The hypothesis is supported by a search of the AceView collection of mouse transcripts, which also contains noncoding RNAs. Although the database covers less than 10% of the ca 2.5 Gb mouse genome, almost one third of our contigs and singletons matching the genome but not ECMT showed similarity to AceView sequences, indicating that the bank vole sequences obtained in the present study are enriched in homologs of sequences transcribed in mouse (the genomes of the mouse and bank vole are of similar size, ca. 2.5 Gb [50]). The finding that, contrary to the situation observed with contigs, more singletons had hits to genome than to EMCT is consistent with the well-known fact that the expression level of most noncoding genome transcripts is generally low and tissue or even cell-type specific [47]. This may also explain the lack of reports of noncoding transcripts in the previous 454 studies of transcriptomes in nonmodel organisms. Either coverage was not sufficient in those studies, or the lack of a moderately divergent model organism, enabling meaningful nucleotide-nucleotide similarity searches against the genome, precluded the identification of noncoding transcripts. Certainly, further experimental studies involving RT-PCR or microarrays would be necessary to validate further our hypothesis and provide more decisive answers as to whether noncoding RNAs indeed represent a substantial portion of the bank vole normalized heart cDNA library.

### SNP differences between selection lines

We identified over 1,000 of putative SNPs that showed apparently significant frequency differences between lines. These polymorphisms constitute an abundant source of candidates for genes underlying microevolutionary response to selection on increased maximum metabolic rate. Overrepresentation of mitochondrial genes among those with SNP frequencies differentiated between selection regimes may be an artifact resulting from generally high coverage of transcripts for mitochondrial proteins in our data. The candidates will be further validated [35,51] and investigated using methods allowing large scale SNP genotyping on an individual basis (reviewed in [52]). The search for genes underlying the response to selection will be facilitated by construction of a genetic map, which has not yet been developed for the bank vole. Single nucleotide polymorphisms and microsatellite markers identified in this study will be useful for this purpose.

## Conclusions

In the present paper, we report the first comprehensive sequence analysis of the bank vole transcriptome. The heart transcriptome was sequenced in the lines selected for high metabolism and in control lines. Longer reads and higher sequence yield per run provided by the 454 Titanium technology proved beneficial for the assembly quality. We detected transcripts of over 14,000 genes, and, for a substantial fraction of them, the full length of coding regions were obtained. Almost full representation of genes known to be expressed in the mouse heart was identified. In addition to genes from the mouse ENSEMBL collection, patterns observed in our data were consistent with widespread transcription from noncoding genomic regions, a finding not reported in previous studies about transcriptomes in non-model organisms. We also detected a number of putative SNPs; a much higher fraction of SNPs than expected by chance exhibited variant frequency differences between selection regimes. These SNPs are thus promising candidates for causal genetic factors underlying response to selection on metabolic rate. The transcript sequences generated in the present study constitute a valuable permanent resource forming a foundation for RNAseq experiments aiming in detection adaptive changes both at the level of gene expression and sequence variants, that would facilitate studies of the genetic basis of evolutionary divergence.

## Methods

### cDNA preparation and 454 sequencing

Four lines selected for a high metabolic rate (4^th ^generation of selection) and four unselected, control lineages were used in the experiment (see [26]). The experimental design and measurement protocols followed internationally recognized guidelines for the research on animals, and were approved by the I Local Ethical Committee for Experiments on Animals in Kraków, according to Polish State Law (permissions number 31/OP/2005 and 99/2006). RNA was extracted from heart tissue of individual voles using the Qiagen RNeasy kit (Qiagen). Equal amounts of RNA from 9-11 individuals per line were combined prior to RNA synthesis. Complementary DNA (cDNA) for each line was synthesized from 1.5-2 μg of RNA using the MINT kit (Evrogen). During the first strand cDNA synthesis primer polTdeg (5' AAGCAGTGGTATCAACGCAGAGTAC (T)_4_G(T)_9_C(T)_10_VN3'; [53]) was used instead of the 3'-primer included in the kit, in order to disrupt the polyA tail of mRNA transcripts. Second cDNA strand was synthesized by PCR amplification. We performed PCR with two primers:

M1ACGG (5'AAGCAGTGGTATCAACGCAGAGTACGG3') and polTM1 (5'AAGCAGTGGTATCAACGCAGAGTAC(T)_4_GTC(T)_4_GTTCTG(T)_3_C(T)_4_VN3'.

M1ACGG was used instead of the M1 primer recommended by the TRIMMER manufacturer, so that it did not anneal to the 5' end of the first strand cDNA containing disrupted polyT sequence. Only polTM1 annealed to this part of cDNA and introduced additional mutations disrupting further polyA/T, such that in double stranded (ds) cDNA only runs of no more than four identical nucleotides were present. Double stranded cDNA was normalized using the TRIMMER kit (Evrogen), equal amounts of normalized cDNA from four selected lines were combined into one pool, and normalized cDNA from four control lines formed the other pool; 5 μg of each pool were sequenced in the separate half of a single 454 Titanium run in the Functional Genomics Center, Uni ⁄ETH Zurich.

### Sequence analysis and assembly

All bioinformatic procedures used publicly available software. Custom Python (BioPython modules) and Perl (BioPerl modules, ENSEMBL API) scripts were used in sequence analysis pipelines. After adapter trimming, we used SeqClean for identifying and removing low complexity regions, overly short reads (shorter than 60 bp), remains of polyA tails, and reads with high similarity to mammalian repetitive sequences in RepBase ver. 14.09. The cleaned sequencing reads produced in this study have been deposited in NCBI's SRA database (accession: SRA012600)

Trimmed reads were searched for microsatellite repeats. Dinucleotide repeats of at least 10 units and tri- and tetranucleotide repeats of at least 8 units long were identified using Msatcommander [54].

Cleaned and trimmed reads were assembled de-novo with the CAP3 Sequence Assembly Program [55]. After preliminary tests, 25 bp overlap and 90% identity were chosen as assembly parameters. All other options of CAP3 were set to default values.

### Functional annotation of the transcriptome

To annotate the transcriptome, we performed similarity searches against both protein and transcriptome/genome databases. A well-annotated general protein database, UniProtKB/Swiss-Prot (ver. 15.10), was searched with BlastX (NCBI BLAST 2.2.20) at an E-value threshold of 10^-5^. The best hit for each contig/singleton was based on the lowest E-value and highest bitscore. If multiple genes produced identical bit scores with a given contig/singleton, ties were broken as follows: i) if exactly one of tied genes gave unambiguous best hit with some other contig(s) or singleton(s), this gene was selected, ii) in the remaining cases ties were broken randomly.

The ENSEMBL collection of mouse transcripts (ECMT, cDNA, ver. NCBIM37.56) was searched with BlastN using an E-value threshold of 10^-5^. If more than one transcript was available for the best hit gene, we conservatively used the longest transcript for downstream analyses. For each result, we assigned Gene Symbol and CDS coordinates, using available ENSEMBL API and custom Perl scripts. Mouse UniGene (Build#182) was also BlastN-searched with an E-value threshold of 10^-5^.

For sequences that did not produce hits in ECMT, we performed a BlastN search against the mouse genome (ENSEMBL DNA ver. NCBIM37.56), rat genome (ENSEMBL DNA ver. NCBIM37.56), and AceView non-redundant mouse transcript base (September 2007), with the same threshold value as above. All results were stored in a MySQL data-base for further data mining.

Using the CORUM Ruepp [56]and BioCyc [57] databases we estimated the completeness of gene discovery for selected macromolecular complexes and basic metabolic pathways which are expected to be present in all nucleated cells

### Completeness of transcripts

To evaluate the completeness of transcripts of genes detected through ECMT similarity searches we used Spidey http://www.ncbi.nlm.nih.gov/spidey/index.html, an mRNA-to-genomic local alignment program. As a reference, we conservatively took the longest transcript (if more than one was available) for each identified mouse gene and aligned all bank vole sequences (contigs and singletons) with significant hits to that gene. In the Spidey analysis, we set inter-species alignment flag to allow for sequence divergence, as the reference was mouse transcripts. Parsing Spidey result files and incorporating information about transcript length and coding sequence CDS location, we computed the fraction of the mouse transcript length covered by the bank vole sequences, both overall and separately for untranslated regions and CDS.

### Identification of SNPs

Single Nucleotide Polymorphisms were identified in GigaBayes http://bioinformatics.bc.edu/marthlab/Software_Release on the basis of CAP3 generated ace files utilizing raw reads and associated quality values. We used the minimum total read coverage for position to be considered (CRL) = 10, minimum read coverage for minor allele (CAL2) = 2, lower probability threshold for reporting polymorphism candidate (PSL) = 0.9; all other parameters were left with default values. The CAL2 = 2 ensures that probability of detecting true SNPs is at least an order of magnitude higher than repeated sequencing errors [e.g., [33]].

## Authors' contributions

WB participated in study design, carried out the laboratory analyses, coordinated the bioinformatic analyses and drafted the manuscript. MS performed bioinformatic analyses and helped to draft the manuscript, WQ helped in bioinformatic analyses, MK performed 454 sequencing, KK participated in the laboratory analyses, PK participated in study design, provided study animals and helped to draft the manuscript, JR conceived of the study, participated in its design and coordination and helped to draft the manuscript. All authors read and approved the final manuscript.

## Supplementary Material

Additional file 1 **Tables S1 and S2**. Supplementary tables in MS Word format Click here for file
