# Supplementary material for: Heart transcriptome of the bank vole (Myodes glareolus): towards understanding the evolutionary variation in metabolic rate
Source: BMC Genomics. 2010 Jun 21;11:390. doi: 10.1186/1471-2164-11-390 (PMC2996923; doi:10.1186/1471-2164-11-390)
Supplement: Additional file 1 — Tables S1 and S2. Supplementary tables in MS Word format [file 1471-2164-11-390-S1.DOC]

**Table S1. Genes most abundantly represented in the normalized bank vole heart transcriptome.**

One hundred contigs with the highest per-base coverage and genes which they represent are listed.

| **Rank** | **Length** | **Coverage** | **Gene symbol** | **Gene full name** |
| --- | --- | --- | --- | --- |
| 1 | 3556 | 2770 | mt-Nd1 | NADH dehydrogenase 1, mitochondrial |
|  |  |  | mt-Rnr2 | 16S rRNA, mitochondrial |
| 2 | 1406 | 1325 | mt-Rnr1 | 12S rRNA, mitochondrial |
| 3 | 940 | 1201 | Nexn | nexilin |
| 4 | 528 | 1162 | Usmg5 | upregulated during skeletal muscle growth 5 |
| 5 | 1135 | 976 | mt-Atp6 | ATP synthase 6, mitochondrial |
|  |  |  | mt-Atp8 | ATP synthase 8, mitochondrial |
|  |  |  | mt-Co3 | cytochrome c oxidase III, mitochondrial |
| 6 | 602 | 912 | Atp5j | ATP synthase, H+ transporting, mitochondrial F0 complex, subunit F |
| 7 | 686 | 900 | Atp5g3 | ATP synthase, H+ transporting, mitochondrial F0 complex, subunit c (subunit 9), isoform 3 |
| 8 | 583 | 850 | Uqcrq | ubiquinol-cytochrome c reductase, complex III subunit VII |
| 9 | 449 | 771 | Cox7c | cytochrome c oxidase, subunit VIIc |
| 10 | 761 | 732 | Ppp1cb | protein phosphatase 1, catalytic subunit, beta isoform |
| 11 | 622 | 697 | Ndufa4 | NADH dehydrogenase (ubiquinone) 1 alpha subcomplex, 4 |
| 12 | 1025 | 693 | mt-Co2 | cytochrome c oxidase II, mitochondrial |
| 13 | 1170 | 674 | Hspa8 | heat shock protein 8 |
| 14 | 512 | 669 | Cox7a2 | cytochrome c oxidase, subunit VIIa 2 |
| 15 | 471 | 627 | Ndufa5 | NADH dehydrogenase (ubiquinone) 1 alpha subcomplex, 5 |
| 16 | 495 | 552 | Cox7b | cytochrome c oxidase subunit VIIb |
| 17 | 6412 | 525 | mt-Cytb | cytochrome b, mitochondrial |
|  |  |  | mt-Nd3 | NADH dehydrogenase 3, mitochondrial |
|  |  |  | mt-Nd4l | NADH dehydrogenase 4L, mitochondrial |
|  |  |  | mt-Nd4 | NADH dehydrogenase 4, mitochondrial |
|  |  |  | mt-Nd5 | NADH dehydrogenase 5, mitochondrial |
|  |  |  | mt-Nd6 | NADH dehydrogenase 6, mitochondrial |
| 18 | 532 | 509 | Atp5j2 | ATP synthase, H+ transporting, mitochondrial F0 complex, subunit f, isoform 2 |
| 19 | 541 | 506 | 1110020P15Rik | RIKEN cDNA 1110020P15 |
| 20 | 554 | 483 | Rps14 | ribosomal protein S14 |
| 21 | 607 | 482 | Lgals1 | lectin, galactose binding, soluble 1 |
| 22 | 530 | 458 | Ndufa6 | NADH dehydrogenase (ubiquinone) 1 alpha subcomplex, 6 (B14) |
| 23 | 474 | 449 | Cox6c | cytochrome c oxidase, subunit VIc |
| 24 | 2776 | 443 | mt-Co1 | cytochrome c oxidase I, mitochondrial |
| 25 | 749 | 423 | Rps8 | ribosomal protein S8 |
| 26 | 441 | 417 | Atp5e | ATP synthase, H+ transporting, mitochondrial F1 complex, epsilon subunit |
| 27 | 550 | 410 | Atp5l | ATP synthase, H+ transporting, mitochondrial F0 complex, subunit g |
| 28 | 541 | 400 | 2310028O11Rik | RIKEN cDNA 2310028O11 |
| 29 | 1684 | 399 | mt-Co3 | cytochrome c oxidase III, mitochondrial |
|  |  |  | mt-Nd3 | NADH dehydrogenase 3, mitochondrial |
|  |  |  | mt-Atp6 | ATP synthase 6, mitochondrial |
| 30 | 1360 | 381 | Pln | phospholamban |
| 31 | 953 | 373 | mt-Nd2 | NADH dehydrogenase 2, mitochondrial |
| 32 | 1261 | 361 | Tnnt2 | troponin T2, cardiac |
| 33 | 670 | 353 | Uqcrb | ubiquinol-cytochrome c reductase binding protein |
| 34 | 940 | 351 | Myl3 | myosin, light polypeptide 3 |
| 35 | 479 | 341 | 2010107E04Rik | RIKEN cDNA 2010107E04 |
| 36 | 821 | 321 | Isca1 | iron-sulfur cluster assembly 1 homolog (S. cerevisiae) |
| 37 | 474 | 319 | Ndufc1 | NADH dehydrogenase (ubiquinone) 1, subcomplex unknown, 1 |
| 38 | 393 | 313 | 2310016M24Rik | RIKEN cDNA 2310016M24 |
| 39 | 491 | 296 | Rpl35 | ribosomal protein L35 |
| 40 | 431 | 290 | Rpl27a | ribosomal protein L27A |
| 41 | 646 | 283 | Atp5h | ATP synthase, H+ transporting, mitochondrial F0 complex, subunit d |
| 42 | 1232 | 281 | Cycs | cytochrome c, somatic |
| 43 | 866 | 269 | Fabp3 | fatty acid binding protein 3, muscle and heart |
| 44 | 409 | 267 | Cox8b | cytochrome c oxidase, subunit VIIIb |
| 45 | 378 | 265 | Eif3h | eukaryotic translation initiation factor 3, subunit H |
| 46 | 667 | 257 | Uqcrh | ubiquinol-cytochrome c reductase hinge protein |
| 47 | 655 | 256 | Cox5b | cytochrome c oxidase, subunit Vb |
| 48 | 1843 | 251 | Ankrd1 | ankyrin repeat domain 1 (cardiac muscle) |
| 49 | 677 | 251 | Mrps18c | mitochondrial ribosomal protein S18C |
| 50 | 1321 | 246 | Ldhb | lactate dehydrogenase B |
| 51 | 584 | 243 | Pgk1 | phosphoglycerate kinase 1 |
| 52 | 369 | 240 | Rps27 | ribosomal protein S27 |
| 53 | 398 | 232 | Cox7a1 | cytochrome c oxidase, subunit VIIa 1 |
| 54 | 706 | 230 | Cox4i1 | cytochrome c oxidase subunit IV isoform 1 |
| 55 | 773 | 225 | Cdh2 | cadherin 2 |
| 56 | 860 | 223 | Myl2 | myosin, light polypeptide 2, regulatory, cardiac, slow |
| 57 | 1192 | 220 | Slc25a3 | solute carrier family 25 (mitochondrial carrier, phosphate carrier), member 3 |
| 58 | 1763 | 219 | Tpm1 | tropomyosin 1, alpha |
| 59 | 521 | 217 | Pgam2 | phosphoglycerate mutase 2 |
| 60 | 451 | 215 | Rpl41 | ribosomal protein L41 |
| 61 | 1425 | 210 | Adprhl1 | ADP-ribosylhydrolase like 1 |
| 62 | 802 | 203 | Ogdh | oxoglutarate dehydrogenase (lipoamide) |
| 63 | 765 | 203 | Eif3f | eukaryotic translation initiation factor 3, subunit F |
| 64 | 851 | 202 | Egln1 | EGL nine homolog 1 (C. elegans) |
| 65 | 688 | 202 | Ndufb9 | NADH dehydrogenase (ubiquinone) 1 beta subcomplex, 9 |
| 66 | 446 | 202 | Ndufa1 | NADH dehydrogenase (ubiquinone) 1 alpha subcomplex, 1 |
| 67 | 378 | 201 | Lmo4 | LIM domain only 4 |
| 68 | 866 | 200 | Nme2 | non-metastatic cells 2, protein (NM23B) expressed in |
| 69 | 903 | 195 | Fth1 | ferritin heavy chain 1 |
| 70 | 1192 | 193 | Tmem182 | transmembrane protein 182 |
| 71 | 453 | 189 | Corin | corin |
| 72 | 599 | 189 | Rcan2 | regulator of calcineurin 2 |
| 73 | 1001 | 185 | Fkbp3 | FK506 binding protein 3 |
| 74 | 2205 | 182 | Sdhb | succinate dehydrogenase complex, subunit B, iron sulfur (Ip) |
| 75 | 1191 | 180 | Rpl7 | ribosomal protein L7 |
| 76 | 1656 | 174 | Nudt4 | nudix (nucleoside diphosphate linked moiety X)-type motif 4 |
| 77 | 531 | 168 | Ndufa2 | NADH dehydrogenase (ubiquinone) 1 alpha subcomplex, 2 |
| 78 | 1300 | 168 | Ces3 | carboxylesterase 3 |
| 79 | 578 | 167 | Ptma | prothymosin alpha |
| 80 | 1020 | 166 | Brp44l | brain protein 44-like |
| 81 | 1836 | 165 | Atp5a1 | ATP synthase, H+ transporting, mitochondrial F1 complex, alpha subunit, isoform 1 |
| 82 | 932 | 164 | Psma7 | proteasome (prosome, macropain) subunit, alpha type 7 |
| 83 | 2806 | 163 | Hsp90aa1 | heat shock protein 90, alpha (cytosolic), class A member 1 |
| 84 | 1409 | 162 | NA | similar to sequence from mouse chromosome 10, probably transcribed as evidenced by EST from mouse UniGene 387999 |
| 85 | 467 | 162 | Usmg5 | upregulated during skeletal muscle growth 5 |
| 86 | 592 | 162 | Ndufv2 | NADH dehydrogenase (ubiquinone) flavoprotein 2 |
| 87 | 468 | 160 | Ndufv3 | NADH dehydrogenase (ubiquinone) flavoprotein 3 |
| 88 | 1696 | 160 | Acaa2 | acetyl-Coenzyme A acyltransferase 2 (mitochondrial 3-oxoacyl-Coenzyme A thiolase) |
| 89 | 886 | 157 | S100a1 | S100 calcium binding protein A1 |
| 90 | 646 | 154 | Shfm1 | split hand/foot malformation (ectrodactyly) type 1 |
| 91 | 477 | 151 | Rplp2 | ribosomal protein, large P2 |
| 92 | 1241 | 151 | Psme4 | proteasome (prosome, macropain) activator subunit 4 |
| 93 | 1089 | 149 | Ak3 | adenylate kinase 3 |
| 94 | 595 | 148 | Ndufa12 | NADH dehydrogenase (ubiquinone) 1 alpha subcomplex, 12 |
| 95 | 633 | 147 | Epdr1 | ependymin related protein 1 (zebrafish) |
| 96 | 517 | 147 | Ndufa13 | NADH dehydrogenase (ubiquinone) 1 alpha subcomplex, 13 |
| 97 | 382 | 146 | Rps29 | ribosomal protein S29 |
| 98 | 1124 | 144 | Atp5c1 | ATP synthase, H+ transporting, mitochondrial F1 complex, gamma polypeptide 1 |
| 99 | 613 | 142 | Tcap | titin-cap |
| 100 | 737 | 141 | Mrps36 | mitochondrial ribosomal protein S36 |

**Table S2. Discovery of genes related to cardiac muscle organization and contraction**

The nonredundant list of mammalian genes in five GeneOntology categories related to cardiac muscle organization and contraction (contractile fiber part (GO00044449), myofibryll (GO0030016), cardiac myofibryll assembly (GO0055003), sacrcomere organisation (GO0045214), cardiac muscle contraction (GO0060048)) detected and not detected in the bank vole transcriptome.

| **Gene symbol** | **Gene full name** |  |
| --- | --- | --- |
| **Detected in the bank vole heart transcriptome** | | |
| Ankrd2 | ankyrin repeat domain 2 (stretch responsive muscle) | |
| Ankrd23 | ankyrin repeat domain 23 | |
| Arf1 | ADP-ribosylation factor 1 | |
| ARF3 | ADP-ribosylation factor 3 | |
| Atp2a1 | ATPase, Ca++ transporting, cardiac muscle, fast twitch 1 | |
| Bmp10 | bone morphogenetic protein 10 | |
| Cacna1c | calcium channel, voltage-dependent, L type, alpha 1C subunit | |
| Cacna1g | calcium channel, voltage-dependent, T type, alpha 1G subunit | |
| CACNA1S | calcium channel, voltage-dependent, L type, alpha 1S subunit | |
| Camk2d | calcium/calmodulin-dependent protein kinase II, delta | |
| Capn3 | calpain 3 |  |
| Capzb | capping protein (actin filament) muscle Z-line, beta | |
| Casq2 | calsequestrin 2 | |
| Cav3 | caveolin 3 |  |
| Ckmt2 | creatine kinase, mitochondrial 2 | |
| Cryab | crystallin, alpha B | |
| Csrp3 | cysteine and glycine-rich protein 3 | |
| Ctgf | connective tissue growth factor | |
| Ctnnb1 | catenin (cadherin associated protein), beta 1 | |
| Dag1 | dystroglycan 1 | |
| Des | desmin |  |
| Dmd | dystrophin, muscular dystrophy | |
| ENO1 | enolase 1, alpha non-neuron | |
| Ep300 | E1A binding protein p300 | |
| Fhl2 | four and a half LIM domains 2 | |
| Fhl3 | four and a half LIM domains 3 | |
| FKBP1B | FK506 binding protein 1b | |
| FLNB | filamin, beta | |
| FLNC | filamin C, gamma | |
| Fxr1 | fragile X mental retardation gene 1, autosomal homolog | |
| Gaa | glucosidase, alpha, acid | |
| Geft | RhoA/RAC/CDC42 exchange factor | |
| Grin2b | glutamate receptor, ionotropic, NMDA2B (epsilon 2) | |
| Hdac4 | histone deacetylase 4 | |
| Homer1 | homer homolog 1 (Drosophila) | |
| Hspb1 | heat shock protein 1 | |
| Ilk | integrin linked kinase | |
| Itgb1 | integrin beta 1 (fibronectin receptor beta) | |
| Itgb1bp2 | integrin beta 1 binding protein 2 | |
| Jph2 | junctophilin 2 | |
| Jup | junction plakoglobin | |
| Kat2b | K(lysine) acetyltransferase 2B | |
| Kcnj8 | potassium inwardly-rectifying channel, subfamily J, member 8 | |
| Krt8 | keratin 8 |  |
| Ldb3 | LIM domain binding 3 | |
| Lman1 | lectin, mannose-binding, 1 | |
| Lrrc10 | leucine rich repeat containing 10 | |
| Map2k3 | mitogen-activated protein kinase kinase 3 | |
| Map2k6 | mitogen-activated protein kinase kinase 6 | |
| Mmp2 | matrix metallopeptidase 2 | |
| Murc | muscle-related coiled-coil protein | |
| MYBPC1 | myosin binding protein C, slow-type | |
| Mybpc3 | myosin binding protein C, cardiac | |
| Myh10 | myosin, heavy polypeptide 10, non-muscle | |
| Myh11 | myosin, heavy polypeptide 11, smooth muscle | |
| MYH13 | myosin, heavy polypeptide 13, skeletal muscle | |
| MYH15 | myosin, heavy chain 15 | |
| Myh3 | myosin, heavy polypeptide 3, skeletal muscle, embryonic | |
| Myh4 | myosin, heavy polypeptide 4, skeletal muscle | |
| Myh6 | myosin, heavy polypeptide 6, cardiac muscle, alpha | |
| Myh7 | myosin, heavy polypeptide 7, cardiac muscle, beta | |
| Myh8 | myosin, heavy polypeptide 8, skeletal muscle, perinatal | |
| Myh9 | myosin, heavy polypeptide 9, non-muscle | |
| Myl1 | myosin, light polypeptide 1 | |
| Myl2 | myosin, light polypeptide 2, regulatory, cardiac, slow | |
| Myl3 | myosin, light polypeptide 3 | |
| MYL4 | myosin, light polypeptide 4 | |
| MYL6B | myosin, light polypeptide 6B | |
| Myl7 | myosin, light polypeptide 7, regulatory | |
| MYL9 | myosin, light polypeptide 9, regulatory | |
| Myo18b | myosin XVIIIb | |
| Myom1 | myomesin 1 | |
| MYOT | myotilin |  |
| Myoz2 | myozenin 2 | |
| Myoz3 | myozenin 3 | |
| MYPN | myopalladin | |
| Nbr1 | neighbor of Brca1 gene 1 | |
| Neb | nebulin |  |
| Neurl2 | neuralized-like 2 (Drosophila) | |
| NOS1 | nitric oxide synthase 1, neuronal | |
| Nrap | nebulin-related anchoring protein | |
| Obscn | obscurin, cytoskeletal calmodulin and titin-interacting RhoGEF | |
| Obsl1 | obscurin-like 1 | |
| P2rx4 | purinergic receptor P2X, ligand-gated ion channel 4 | |
| Pak1 | p21 protein (Cdc42/Rac)-activated kinase 1 | |
| Palld | palladin, cytoskeletal associated protein | |
| Pdlim3 | PDZ and LIM domain 3 | |
| Pdlim5 | PDZ and LIM domain 5 | |
| Pecam1 | platelet/endothelial cell adhesion molecule 1 | |
| Pgm5 | phosphoglucomutase 5 | |
| Ppp3ca | protein phosphatase 3, catalytic subunit, alpha isoform | |
| Prox1 | prospero-related homeobox 1 | |
| Pygm | muscle glycogen phosphorylase | |
| Ryr1 | ryanodine receptor 1, skeletal muscle | |
| Ryr2 | ryanodine receptor 2, cardiac | |
| Sdc4 | syndecan 4 | |
| Sfrs1 | splicing factor, arginine/serine-rich 1 (ASF/SF2) | |
| Slc4a1 | solute carrier family 4 (anion exchanger), member 1 | |
| Smad5 | MAD homolog 5 (Drosophila) | |
| Smad7 | MAD homolog 7 (Drosophila) | |
| SORBS2 | sorbin and SH3 domain containing 2 | |
| Spna2 | spectrin alpha 2 | |
| SPTBN1 | spectrin, beta, non-erythrocytic 1 | |
| Sri | sorcin |  |
| Svil | supervillin |  |
| Sync | syncoilin |  |
| Syne1 | synaptic nuclear envelope 1 | |
| Syne2 | synaptic nuclear envelope 2 | |
| Synpo2 | synaptopodin 2 | |
| Taz | tafazzin |  |
| Tcap | titin-cap |  |
| Timp4 | tissue inhibitor of metalloproteinase 4 | |
| Tmod1 | tropomodulin 1 | |
| Tnnc1 | troponin C, cardiac/slow skeletal | |
| Tnni3 | troponin I, cardiac 3 | |
| Tnnt1 | troponin T1, skeletal, slow | |
| Tnnt2 | troponin T2, cardiac | |
| Tnnt3 | troponin T3, skeletal, fast | |
| Tpm1 | tropomyosin 1, alpha | |
| Tpm2 | tropomyosin 2, beta | |
| TPM3 | tropomyosin 3, gamma | |
| TPM4 | tropomyosin 4 | |
| Trim32 | tripartite motif-containing 32 | |
| Trim54 | tripartite motif-containing 54 | |
| Trpc1 | transient receptor potential cation channel, subfamily C, member 1 | |
| Ttn | titin |  |
| Vcl | vinculin |  |
| Vegfb | vascular endothelial growth factor B | |
| Xirp1 | xin actin-binding repeat containing 1 | |
| **Not detected in the bank vole heart transcriptome, absent in the mouse or rat heart EST database** | | |
| Cxcr4 | chemokine (C-X-C motif) receptor 4 | |
| Jph1 | junctophilin 1 | |
| Myh1 | myosin, heavy polypeptide 1, skeletal muscle, adult | |
| Myh2 | myosin, heavy polypeptide 2, skeletal muscle, adult | |
| MYL5 | myosin, light chain 5, regulatory | |
| MYLK2 | myosin, light polypeptide kinase 2, skeletal muscle | |
| Mylpf | myosin light chain, phosphorylatable, fast skeletal muscle | |
| Psen2 | presenilin 2 | |
| Smtnl1 | smoothelin-like 1 | |
| Tnnc2 | troponin C2, fast | |
| **Not detected in the bank vole heart transcriptome, present in the mouse or rat heart EST database** | | |
| Edn1 | endothelin 1 | |
| Krt19 | keratin 19 |  |
| Nkx2-5 | NK2 transcription factor related, locus 5 (Drosophila) | |
| Synm | synemin, intermediate filament protein | |
| Tnni1 | troponin I, skeletal, slow 1 | |
| Tnni2 | troponin I, skeletal, fast 2 | |
